# Tumor suppressor OTUD3 induces growth inhibition and apoptosis by directly deubiquitinating and stabilizing p53 in invasive breast carcinoma cells

**DOI:** 10.1186/s12885-020-07069-9

**Published:** 2020-06-22

**Authors:** Qian Pu, Yan-rong Lv, Ke Dong, Wen-wen Geng, Hai-dong Gao

**Affiliations:** 1 Department of General Surgery, Qilu Hospital of Shandong University, Jinan, Shandong 250012 P.R. China; 2 Department of General Surgery, Qilu Hospital (Qingdao) of Shandong University, 758 Hefei Road, Qingdao, Shandong 266035 P.R. China; 3 Shandong University, Jinan, Shandong 250012 P.R. China

**Keywords:** OTUD3, p53, Deubiquitinating enzymes, Invasive breast carcinoma

## Abstract

**Background:**

P53 pathway inactivation plays an important role in the process of breast cancer tumorigenesis. Post-translational protein modification abnormalities have been confirmed to be an important mechanism underlying inactivation of p53. Numerous deubiquitinating enzymes are aberrantly expressed in breast cancer, and a few deubiquitination enzymes can deubiquitinate and stabilize p53. Here, we report that ovarian tumor (OTU) deubiquitinase 3 (OTUD3) is a deubiquitylase of p53 in breast carcinoma (BC).

**Methods:**

Correlations between the mRNA expression levels of OTUD3, TP53 and PTEN and the prognosis of BC were assessed with the Kaplan-Meier Plotter tool. OTUD3 protein expression in 80 pairs of specimens in our cohort was examined by immunohistochemistry and western blotting. The relationship among OTUD3, p53, and p21 proteins was analyzed. Half-life analysis and ubiquitylation assay were performed to elucidate the molecular mechanism by which OTUD3 stabilizes p53. The interaction between OTUD3 and p53 in BC cells was verified by a co-immunoprecipitation assay and GST pulldown experiments. MTS assay for proliferation detection, detection of apoptosis induced by cisplatin and colony formation assay were employed to investigate the functional effects of OTUD3 on breast cancer cells.

**Results:**

OTUD3 downregulation is correlated with a poor prognosis in BC patients. OTUD3 expression is decreased in breast cancer tissues and not associated with the histological grade. OTUD3 also inhibits cell proliferation and clone formation and increases the sensitivity of BC cells to apoptosis induced by chemotherapy drugs. Reduced OTUD3 expression accompanied by decreased p53 abundance is correlated with human breast cancer progression. Ectopic expression of wild-type OTUD3, but not its catalytically inactive mutant, stabilizes and activates p53. Mechanistically, OTUD3 interacts directly with p53 through the amino-terminal OTU region. Finally, OTUD3 protects p53 from murine double minute 2 (Mdm2)-mediated ubiquitination and degradation, enabling the deubiquitination of p53 in BC cells.

**Conclusions:**

In summary, we found that OTUD3 may be a potential therapeutic target for restoring p53 function in breast cancer cells and suggest that the OTUD3-p53 signaling axis may play a critical role in tumor suppression.

## Background

Invasive breast carcinoma (BC) is the leading cause of all new cancer diagnoses in women [1]. Tumor protein p53 pathway inactivation plays an important role in the process of BC tumor genesis. Wild-type p53 is present in approximately 70% of BC cases [2], and the p53 pathway is partially abrogated through inactivation of various signals or effector elements [3]. Accordingly, the role of p53 signaling in BC tumorigenesis has attracted considerable attention [4]. In addition to point mutations and gene deletions, post-translational protein modification abnormalities have been confirmed to be an important mechanism underlying inactivation of p53. Among these abnormalities, ubiquitination is more complex than phosphorylation and acetylation [5]. Most research focuses on the regulatory effect of ubiquitin ligase on the ubiquitination of p53 [6]; such research includes the well-known ubiquitination enzyme murine double minute 2 (Mdm2) [7, 8]. Recently, the N-terminal p53 TAD and Mdm2 pBD regions were studied to discover anticancer drug molecules [4]. However, limited success was achieved due to tumor recurrence [9] or *TP53* gene mutations [10]. The intriguing nature of the regulation of p53 signaling and its role in tumorigenesis are certainly perplexing due to the complexity involved [4]. Therefore, identifying more strategies to stabilize p53 is particularly important.

The ubiquitination of many proteins has been well documented to be reversed by deubiquitinating enzymes (DUBs), which belong to a superfamily of cysteine proteases and metalloproteases that cleave ubiquitin-protein bonds. The human genome encodes approximately 100 DUBs [11] that can be classified into the following six families: ubiquitin-specific proteases (USPs), ubiquitin car boxy-terminal hydrolases (UCHs), ovarian tumor (OTUs) proteases, Machado-Joseph disease protein domain proteases (MJDs), JAMM/MPN domain-associated metallopeptidases (JAMMs), and monocyte chemotactic protein-induced proteins (MCPIPs).

In BC, numerous DUBs [11], including breast cancer-promoting DUBs and cancer-suppressing DUBs, are aberrantly expressed. However, only two deubiquitination enzymes can deubiquitinate and stabilize p53 [11],and USP7 (HAUSP) might represent the first example [12]. However, TSPYL5 can bind USP7 and suppress its ability to deubiquitinate and stabilize p53 [13]. In addition, an interesting feedback loop exists in p53 regulation because USP7 also binds, deubiquitinates and stabilizes Mdm2 more potently under physiologic conditions [14, 15] and stabilizes p53 under genotoxic stress conditions [16, 17]. USP10 can deubiquitinate cytoplasmic p53 and inhibit MDM2-mediated p53 nuclear export and degradation. USP10 can also shuttle into the nucleus and stabilize p53 when DNA damage occurs [18]. However, USP10 may stabilize both wild-type p53 and mutant p53 [19] and is more highly expressed in breast cancer tissue than in adjacent normal tissue [20]. Unsurprisingly, such an important tumor suppressor is controlled by multiple DUBs. However, few DUBs have been found in breast cancer, and the mechanisms regulating p53 deubiquitination remain enigmatic.

Our previous study found that OTU deubiquitinase 3 (OTUD3) can deubiquitinate and stabilizes PTEN [21]. In the current study, we found that the expression of OTUD3 was decreased in BC and proved for the first time that OTUD3 is an enzyme related to the deubiquitination of p53. Compared with PTEN, high expression levels of OTUD3 and p53 are more indicative of a better prognosis in BC. This study further elucidated the influence of OTUD3 on BC cell biological function and its molecular mechanism and suggests that OTUD3 should be explored as a therapeutic target in breast cancer.

## Methods

### Kaplan-Meier plotter

Correlations between the mRNA expression levels of *OTUD3, TP53* and *PTEN* and the prognosis of BC were assessed with the Kaplan-Meier Plotter tool [22, 23] (http://kmplot.com/analysis/index). BC patients were divided into two groups according to median expression levels (high expression vs. low expression). A Kaplan-Meier survival chart was used in the analysis to evaluate the relapse-free survival (RFS) of the patients, and the risk ratio (HR) and its 95% confidence interval (CI) and the log- rank test were used to calculate the *p*-value.

### Cells and tumor tissues

Two human breast cancer cell lines, MCF-7 and DU4475, were obtained from the American Type Culture Collection (ATCC, Manassas, VA, USA). The MCF7 cells were cultured at 37 °C in Dulbecco’s modified Eagle’s medium (DMEM) supplemented with 10% FBS (HyClone, USA) under a 5% CO_2_ atmosphere. The DU4475 cells were cultured in RPMI-1640 medium supplemented with 10% FBS. This study was approved by the Human Ethics Review Committee of Qilu Hospital (Qingdao) of Shandong University. The use of eighty paired breast cancer tissues and matched adjacent normal tissues was approved by the Department of Pathology of Qilu Hospital (Qingdao) of Shandong University. All patients underwent surgical resection at Qilu Hospital (Qingdao) of Shandong University. Informed consent was obtained from all subjects or their relatives.

### Antibodies and reagents

An anti-OTUD3 antibody (HPA028544) for immunohistochemistry (IHC) and the proteasome inhibitor MG132 were purchased from Sigma-Aldrich, USA. An anti-OTUD3 antibody (ab107646), wild-type anti-p53 antibody (ab131442), and anti-p21 antibody (ab109520) for western blotting were purchased from Abcam, United Kingdom. An anti-glyceraldehyde 3-phosphate dehydrogenase antibody (anti-GAPDH) and secondary antibodies were purchased from Santa Cruz Biotechnology, Inc., USA. Anti-Myc and anti-Flag antibodies were obtained from MBL, BEIJING B&M BIO TECH CO.,LTD, Beijing, China.

### Immunohistochemistry

IHC was performed by using the avidin-biotin complex method, including heat-induced antigen-retrieval procedures. Incubation with an antibody against OTUD3 (1:100 dilution; HPA028544) was carried out at 4 °C for 18 h. All staining was assessed by a quantitative imaging method (inForm, PerkinElmer) utilizing continuous measurement and pathologists blinded to the sample origins and subject outcomes. The widely accepted German semi-quantitative scoring system based on the staining intensity and area was used. Each specimen was assigned a score according to the intensity of nuclear, cytoplasmic, and/or membrane staining (no staining = 0; weak staining = 1, moderate staining = 2, and strong staining = 3) and the extent of stained cells (0% = 0, 1–24% = 1, 25 = 49% = 2, 50–74% = 3, and 75–100% = 4). The final immunoreactive score was determined by multiplying the intensity score by the extent score and ranged from 0 (minimum) to 12 (maximum).

### Lentivirus infection

Lentiviruses carrying shRNA targeting human OTUD3 lentiviral vectors (GV112) were obtained from GeneChem. We constructed lentiviruses carrying overexpression lentiviral vectors. The viruses were used to infect cells in the presence of polybrene. After forty-eight hours, MCF7 or DU4475 cells were cultured in medium containing puromycin for the selection of stable clones. The clones with stable OTUD3 knockdown were identified and verified by western blotting. The shRNA sequences were as follows: OTUD3 no. 1: 5′-TGGAAATCAGGGCTTAAAT-3′; no. 2, 5′-GAGTTACACATCGCATATC-3′; no. 3, 5′-CGTCTGCCATCGCATATTA-3′; and non-targeting control, 5′-TTCTCCGAACGTGTCACGT-3′.

### Western blot (WB) analysis

The cells and tissue specimens were lysed using RIPA buffer (Sigma-Aldrich, St. Louis, MO, USA). The protein samples were separated using 10% sodium dodecyl sulfate-polyacrylamide gel electrophoresis (SDS-PAGE) and transferred onto PVDF membranes (Millipore). The membranes were blocked with 5% non-fat milk at room temperature for 2 h and incubated overnight with primary antibodies at 4 °C. After washing with TBST three times for 15 min each, the membranes were incubated with the appropriate horseradish peroxidase-conjugated secondary antibodies for 2 h at room temperature with slight shaking. GAPDH was used as the loading control. The immunoreactive bands were visualized using SuperSignal West Pico Chemiluminescent Substrates (Thermo Fisher Scientific, USA).

### Protein half-life assay

For the p53 half-life assay, the MCF7 and DU4475 cells were grown in 2-cm plates to approximately 60% confluence, and then the cells were transfected with OTUD3 shRNAs. After twenty-four hours, the cells were treated with the protein synthesis inhibitor cycloheximide (CHX, Sigma, 10 μg ml-1) for the indicated durations before collection.

### Immunoprecipitation

The cultured cells were lysed with HEPES lysis buffer (20 mM HEPES, pH 7.2, 50 mM NaCl, 0.5% Triton X-100, 1 mM NaF and 1 mM dithiothreitol) supplemented with Protease Inhibitor Cocktail Tablets (Roche). The immunoprecipitations were performed using the indicated primary antibody and protein A/G agarose beads (Santa Cruz) at 4 °C. Then, the immunocomplexes were washed with HEPES lysis buffer four times. Both the lysates and immunoprecipitates were examined using the indicated primary antibodies, followed by incubation with the appropriate secondary antibody and SuperSignal West Pico Chemiluminescent Substrate (Thermo Fisher Scientific).

### GST pulldown assays

Bacterial-expressed GST and GST-p53 bound to glutathione-Sepharose 4B beads (from GE) were incubated with Myc-OTUD3-expressing MCF7 cells for 2 h at 4 °C. Then, the beads were washed with GST binding buffer (100 mM NaCl, 10 mM Tris,50 mM NaF, 2 mM EDTA, 0.5 mM Na_3_VO_4_ and 1% Nonidet P40) four times, and the proteins were eluted and subjected to western blotting.

### Ubiquitylation assay

The cells were treated with 20 mM MG132 proteasome inhibitor for 8 h. Then, the cells were washed with PBS and lysed in 0.5 ml of HEPES buffer (20 mM HEPES, pH 7.2, 50 mM NaCl, 1 mM NaF, and 0.5% Triton X 100) supplemented with 0.1% SDS and a protease inhibitor cocktail (Roche, Germany). The lysates were centrifuged to obtain the cytosolic proteins. Briefly, the individual samples were incubated with primary antibodies for 3 h, followed by incubation with protein A/G agarose beads (Santa Cruz) for another 8 h at 4 °C. The beads were washed three times with HEPES buffer. The proteins were released from the beads by boiling in 40 ml of 26SDS-PAGE sample buffer for 10 min. The samples were subjected to a WB analysis.

### Proliferation assay

The cells were plated in 96-well plates (100-μl cell suspensions, 1*10^4^ cells ml^− 1^) and assayed for MTS (3-(4, 5-dimethylthiazol-2-yl)-5-(3-carboxymethoxyphenyl)-2-(4-sulphophenyl)-2H-tetrazolium, inner salt, Sigma) reduction. Twenty-four hours after plating, 0.05 mg ml-^1^ MTS reagent (Promega) was added to each well, and the cells were incubated at 37 °C for 4 h, followed by absorbance measurement at 490 nm. The sample values were standardized to those of wells containing medium alone.

### Apoptosis assays

First,cells were treated with cisplatin (10 mM, 24 h, Sigma-Aldrich, USA). After incubation, the cells were washed with PBS and stained with fluorescein isothiocyanate-Annexin V and propidium iodide according to the manufacturer’s protocol (Beijing Biosea Biotechnology Annexin V Kit). Then, the apoptotic cells (Annexin V-positive, propidium iodide-negative) were determined by flow cytometry.

### Colony formation assays

Cells were resuspended in DMEM containing 0.35% low-melting agarose (Sigma) and 10% FBS and seeded onto a coating of 0.7% low-melting agarose in DMEM containing 10% FBS. The plates were incubated at 37 °C and 5% CO_2_, and the colonies were scored 3 weeks after preparation. Colonies larger than 0.1 mm in diameter were scored as positive.

### Statistical analysis

Differences between two independent groups were evaluated using an unpaired Student’s t-test. Chi-square tests and one-way ANOVA were used to compare the groups. Correlation analysis was performed using Spearman’s rank correlation coefficient. All IHC and WB statistical analyses were performed with GraphPad Prism 7.00 and SPSS 19.0(IBM Corp, USA). All other results are expressed as the mean ± standard deviation (SD) of three independent experiments unless stated otherwise. All statistical tests were two-sided, and *p*-values< 0.05 (*) or < 0.01 (**) were considered statistically significant.

## Results

### High OTUD3 expression is downregulated in BC tissues and associated with a better prognosis in BC patients

**Fig. 1:**
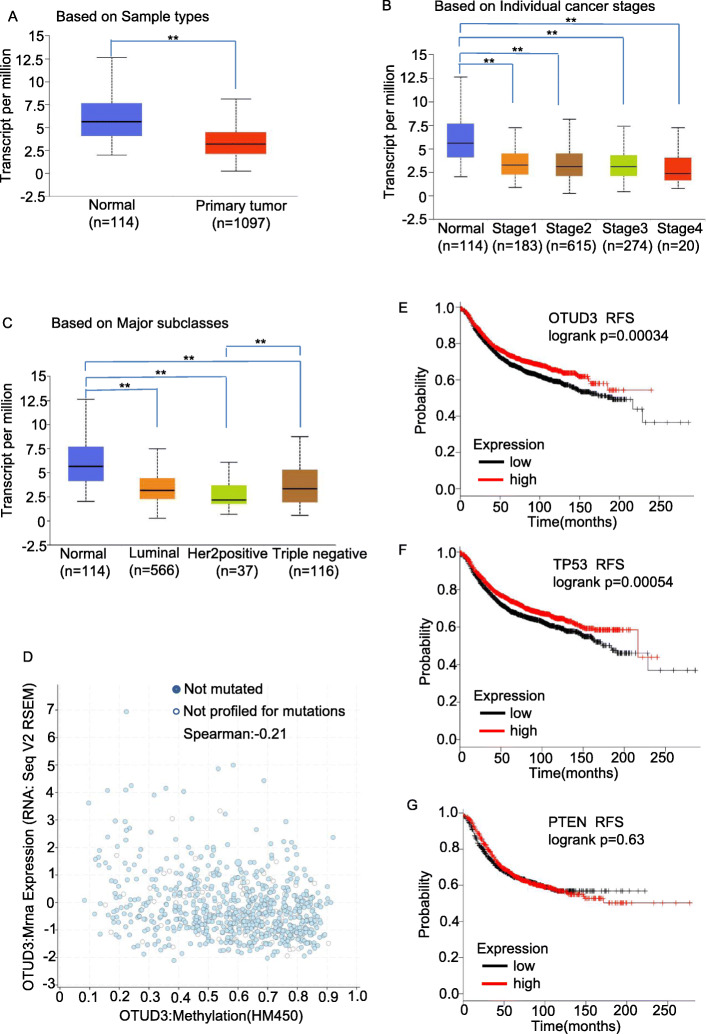
OTUD3 downregulation is correlated with a poor prognosis in BC patients. **a** The transcriptional level of OTUD3 in BC tissues compared to that in normal breast tissues. **b**-**c** The transcriptional level of OTUD3 in BC patients based on individual cancer stages and major subclasses. **d**. Correlation scatter plot of OTUD3 mRNA expression and methylation levels in BC patients. **e**-**g** Prognostic value of the mRNA levels of OTUD3, p53 and PTEN in BC patients (RFS according to the Kaplan-Meier Plotter). **e** OTUD3 (213216_s_at). **f** P53 (201746_at). **g** PTEN (225363_at)

OTUD3 is not frequently mutated in the TCGA pan-cancer dataset (https://www.cbioportal.org/). To assess OTUD3 expression in BC patients, we first analyzed the gene expression UALCAN database [24] (datasetshttp://ualcan.path.uab.edu/)for human BC. The results showed that OTUD3 mRNA levels in BC tissues were significantly lower than those in normal breast tissues (Fig. 1a). *OTUD3* mRNA levels were not associated with individual cancer stages (Fig. 1b). Although no differences in OTUD3 mRNA levels were found between luminal samples and HER2-positive or triple-negative BC samples, a difference in OTUD3 mRNA levels was identified between the HER2-positive and triple-negative BC samples (Fig. 1c). We used data obtained from the cBioPortal database [25, 26] (https://www.cbioportal.org/) and found that decreases in OTUD3 mRNA levels may not be due to increased OTUD3 DNA methylation in BC (Fig. 1d).

Our previous results showed that OTUD3 can deubiquitinate and stabilize PTEN. Here,we explored whether OTUD3 is an enzyme related to p53.Therefore, we performed a Kaplan-Meier survival analysis [22, 23] (http://kmplot.com/analysis/index) to evaluate the associations between OTUD3, TP53 and PTEN expression and survival in BC patients. Interestingly, the recurrence-free survival (RFS) rate in the high-OTUD3 expression group (*n* = 1986) was better than that in the low-OTUD3 expression group (*n* = 1965), *p* = 0.00034 (Fig. 1e). Similarly, patients with high *TP53* (*n* = 1977) expression had better RFS than the patients with low expression (*n* = 1974), *p* = 0.00054 (Fig. 1f). However, the expression level of *PTEN* was not an independent prognostic factor in the BC patients in the dataset, *p* = 0.62 (Fig. 1g). We speculate that high OTUD3 expression is associated with a better prognosis in BC patients, and that the relationship between OTUD3 and p53 is the most significant.

### OTUD3 expression is downregulated in BC tissue

**Fig. 2:**
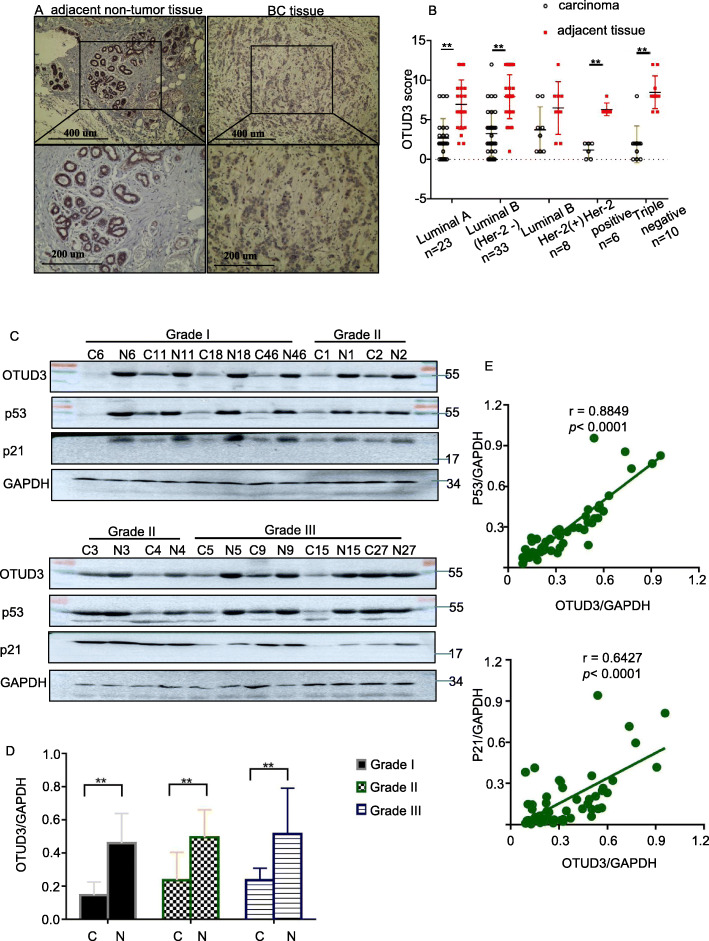
OTUD3 expression is decreased in breast cancer tissues. **a** Representative IHC staining images of OTUD3 in BC tissues and normal breast tissues. The magnifications of the upper and lower images are 100× and 200×, respectively. **b** OTUD3 staining was assessed, calculated, plotted and analyzed. **p < 0.01 (Chi-square test). **c** WB results of 12 pairs of fresh tissues samples. **d** OTUD3 expression was independent of the histological grade. **e** Positive correlations were identified among OTUD3, p53 and p21 expression in breast cancer tissues. C, breast cancer tissue; N, matched normal tissue

Consistent with the OTUD3 mRNA results, the protein expression level of OTUD3 was also significantly lower in BC tissue than in normal breast tissue. IHC staining with an anti-OTUD3 antibody was performed on 80 pairs of BC tissues and adjacent non-tumor tissues. Staining scores of 0–6 were considered negative, and scores of 6–12 were considered positive. As shown in Fig. 2a, substantial OTUD3 immunostaining was detected in the adjacent non-tumor tissue samples, whereas little to moderate OTUD3 staining was observed in the BC samples. According to the literature [27], BC was divided into the following subtypes: luminal A-like, luminal B-like (Her-2-negative), luminal B-like (Her-2-positive), Her-2 positive (non-luminal) and triple-negative (ductal). Notably, OTUD3 expression in breast cancer tissue was not associated with the molecular type (χ^2^ = 2.672,*p =* 0.614)(Fig. 2b). Although not statistically significant, a trend indicating that OTUD3 may be downregulated in luminal B (Her-2-positive) cancer tissue compared with adjacent tissue was observed.

The half-life of the wild-type p53 protein is very short, and the p53 protein detected by IHC in various experiments is the mutant p53 protein [28, 29]. Therefore, we analyzed the expression of p53 and OTUD3 in 26 pairs of fresh and frozen BC tissues and adjacent normal tissues by WB. The expression of the p53 downstream protein p21 was also detected. *P21*^*WAP1/cip1*^ is a p53-induced cell cycle kinase inhibitor (CDKI) [30]. P53 causes G1 cell arrest by regulating the expression of p21 [31]. Knockout of p21 results in complete loss of p53-mediated human tumor cell cycle(G1) arrest [32]. The specimens were numbered by the date of collection and grouped by histological grade. Our experimental results prove for the first time that OTUD3 and p53 are both expressed in breast cancer and adjacent normal tissues.

Notably, the expression of OTUD3(*p* = 0.0069) and p53(*p* = 0.041) in all BC tissues was lower than that in the corresponding normal tissues (Fig. 2c). OTUD3 expression in breast cancer tissue was independent of the histological grade (F = 1.736, *p* = 0.199) (Fig. 2d). Additionally, the analysis of the relationship between OTUD3 expression and p53 expression showed a significantly positive correlation (r = 0.8849, 95% CI: 0.8068–0.9326, *p* < 0.0001), and the levels of OTUD3 and p21 were also positively correlated (r = 0.6427, 95% CI: 0.4484–0.779, *p* < 0.0001) (Fig. 2e). Our clinical data fully proved that OTUD3 is downregulated in cancer tissues and is highly correlated with p53 expression.

### OTUD3 maintains p53 stability in vitro

**Fig. 3:**
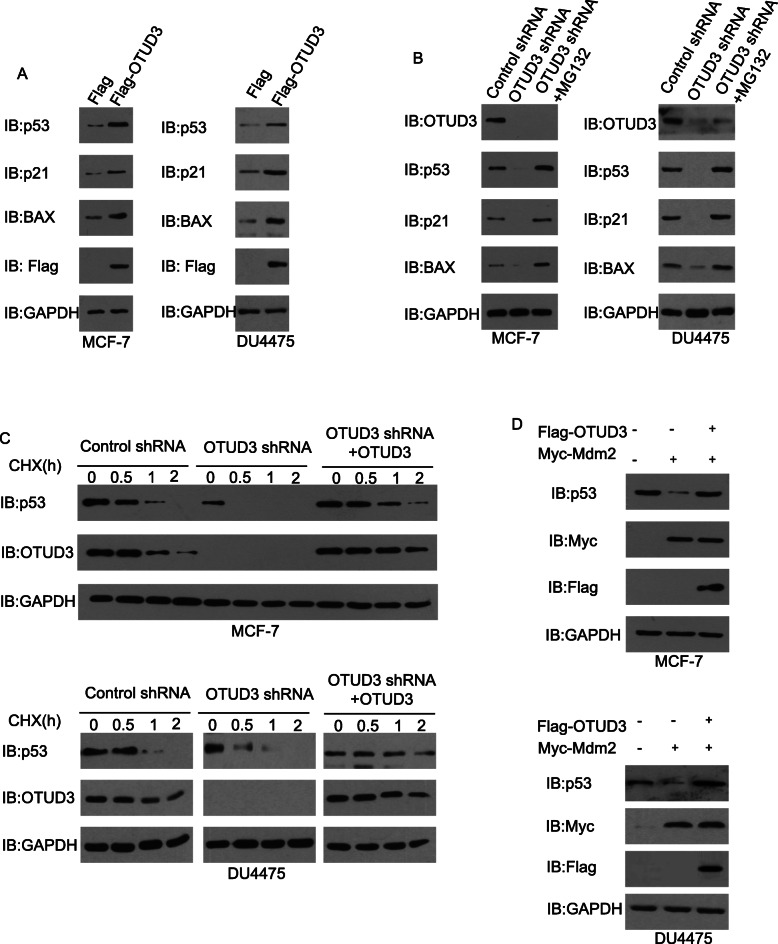
OTUD3 stabilizes p53. **a** MCF7 cells and DU4475 cells transfected with an OTUD3-expressing vector or control (Flag) vector were analyzed to detect the indicated proteins by immunoblotting (IB). **b** MCF7 cells and DU4475 cells transfected with sh-ctrl or sh-OTUD3 lentivirus were treated with or without the proteasome inhibitor MG132 (20 μM, 8 h) and then analyzed to detect the indicated proteins. **c** Half-life analysis of p53 in MCF7 cells and DU4475 cells. **d** Protection of p53 from Mdm2-mediated degradation by OTUD3 in BC cells

Since the expression levels of OTUD3 and p53 are correlated we tested whether overexpression of OTUD3 affects p53 protein levels in breast cancer cells. Two well-established p53 target genes, p21 and the proapoptotic gene BCl-2-associated X protein (BAX), were assayed to reflect p53 activity. Here, we used the luminal breast cancer cell line MCF7 and the TNBC cell line DU4475, both of which express wild-type p53 [33]. As shown in Fig. 3a, the p53 level was dramatically increased when *OTUD3* was overexpressed in the MCF7 cells and DU4475 cells; increased p21 and BAX protein levels were also observed, indicating that OTUD3 also causes p53-dependent transcriptional activation. To confirm the role of OTUD3 in the regulation of p53, the MCF7 and DU4475 cells were transfected with sh-ctrl or sh-OTUD3 lentivirus. Changes in the p53 protein level were determined. We found that *OTUD3* knockdown in BC cells resulted in a dramatic decrease in the protein level of endogenous p53,which was accompanied by a decrease in the two target genes (Fig. 3b). To test the possibility that OTUD3 regulation of p53 occurs through modulation of p53 protein stability, we treated cells stably expressing *OTUD3* shRNA with the proteasome inhibitor MG132 (20 μM, 8 h). The decrease in p53 levels was reversed by MG132 treatment (Fig. 3b), suggesting that OTUD3 regulates p53 levels by increasing its stability in a proteasome-dependent manner.

Ubiquitin-mediated degradation is the only method by which p53 is terminated by the proteasome [34]. To date, some p53 E3 ligases have been found, and MDM2 is the most important [35]. MDM2 (human HDM2) is an oncogene that promotes cell division and proliferation [36]. MDM2 mediates the sustainable degradation of most p53 proteins, resulting in a very low p53 intracellular level [37].P53 is regulated by Mdm2-mediated ubiquitination and degradation and has a short half-life (5–20 min) [38]. Subsequently, we examined the half-life of p53 in the absence or presence of OTUD3. We treated control cells and cells stably expressing *OTUD3* shRNA with or without OTUD3 overexpression with the protein synthesis inhibitor cycloheximide (CHX) and examined p53 levels at various time points. The half-life of endogenous p53 was significantly shortened in the BC cells depleted of *OTUD3*, and this effect was fully reversed by the ectopic expression of OTUD3 (Fig. 3c). OTUD3 likely deubiquitinates p53 to counteract the action of the E3 ubiquitin ligase Mdm2. Indeed, as shown in Fig. 3d, ectopic *Mdm2* expression significantly induced p53 degradation, while the coexpression of *OTUD3* efficiently rescued p53 from Mdm2-induced degradation. These results demonstrate that OTUD3 can antagonize the reduction in p53 by Mdm2.

### OTUD3 interacts with p53

**Fig. 4:**
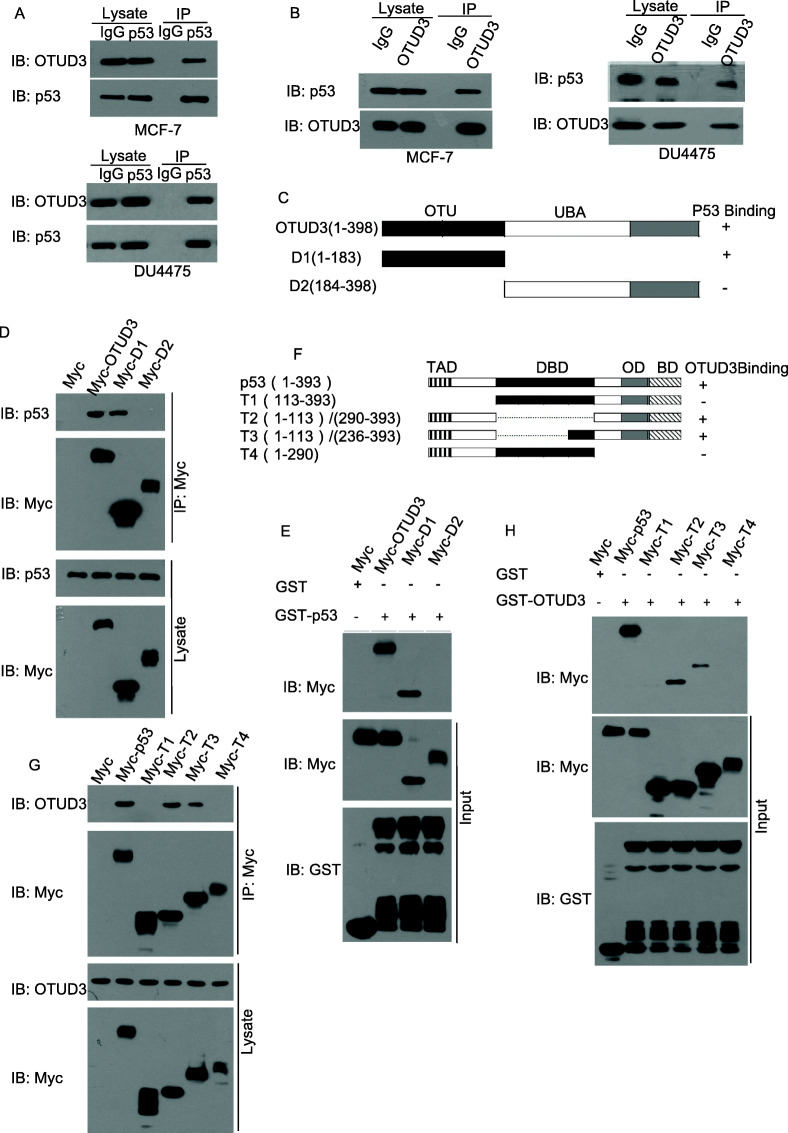
OTUD3 interacts with p53. **a**-**b** Co-immunoprecipitation of endogenous OTUD3 and p53 proteins in MCF7 and DU4475 cells. MCF7 and DU4475 cell extracts were immunoprecipitated with anti-p53 (**a**) or anti-OTUD3 (**b**) antibodies, and rabbit IgG served as a negative control. **c** Overview of the OTUD3 structure and diagrams of the D1 and D2 constructs. A summary of their binding status with p53 is shown on the right. **d** Extracts of MCF7 cells transfected with the indicated constructs were subjected to immunoprecipitation with an anti-Myc antibody, followed by western blot analysis with the indicated antibodies. **e** A direct interaction between GST-p53 and OTUD3 and its truncated mutant was detected by GST pulldown experiments. **f** Overview of the p53 structures. A summary of their binding status with OTUD3 is shown on the right. **g** A Myc antibody was used for IP, and IB was performed to detect the interaction between OTUD3 and p53 and their truncated mutants in the co-IP experiment. **h** GST pulldown experiments were performed to detect the direct interaction between GST-OTUD3 and p53 and its truncated mutant

Co-immunoprecipitation (CO-IP) assays were conducted in MCF7 cells and DU4475 cells to examine whether OTUD3 physically interacts with p53. As shown in Fig. 4a, endogenous OTUD3 was specifically co-immunoprecipitated with endogenous p53 in the cells by anti-p53 antibodies. Furthermore, p53 co-immunoprecipitated with endogenous OTUD3 in the MCF7 cells and DU4475 cells (Fig. 4b). Then, we constructed OTUD3 truncated mutants as follows: OTUD3 was divided into two parts per its domain structure, namely, D1 (1–183) containing the OTU domain and D2 (184–398) containing the UBA domain and the C tail (Fig. 4c). Using the D1 and D2 constructs in co-immunoprecipitation experiments, we revealed that the OTU domain-containing region D1 (1–183) is critical for the interaction between OTUD3 and p53 (Fig. 4d). To determine whether the OTUD3-TP53 interaction was direct, we generated and purified recombinant Myc-OTUD3, Myc-D1 and Myc-D2. Interestingly, Myc-D1 cultured from MCF7 cells was specifically bound by the purified GST-TP53 protein but not GST alone (Fig. 4e), further illustrating the direct interaction between OTUD3 and p53.

*TP53* is located on chromosome 17 (17p13.1) and encodes p53, which is a phosphoprotein comprising 393 amino acids. P53 consists of the following four domains: (I) an N-terminal sequence (transactivation domain, TAD) involved in the regulation of target gene transcription, recruitment of RNA polymerase and activation of the transcriptional (DNA-reading) machinery, (II) a highly conserved DNA-binding domain (DBD) that recognizes specific DNA sequences; (III) an oligomerization domain (OD) that assembles chains of other p53 monomers for tetramerization, and (IV) a C-terminal domain essential for the regulation of p53 activity. To determine the region where p53 binds OTUD3, we also constructed truncated p53 mutants (Fig. 4f) and performed GST assays. The results indicated that OTUD3 bound the following two mutants of p53: T2 (1–113)/(290–393) and T3 (1–113)/(236–393) (Fig. 4g, h). These results suggest that a direct interaction exists between OTUD3 and p53.

### OTUD3 deubiquitinates p53

**Fig. 5:**
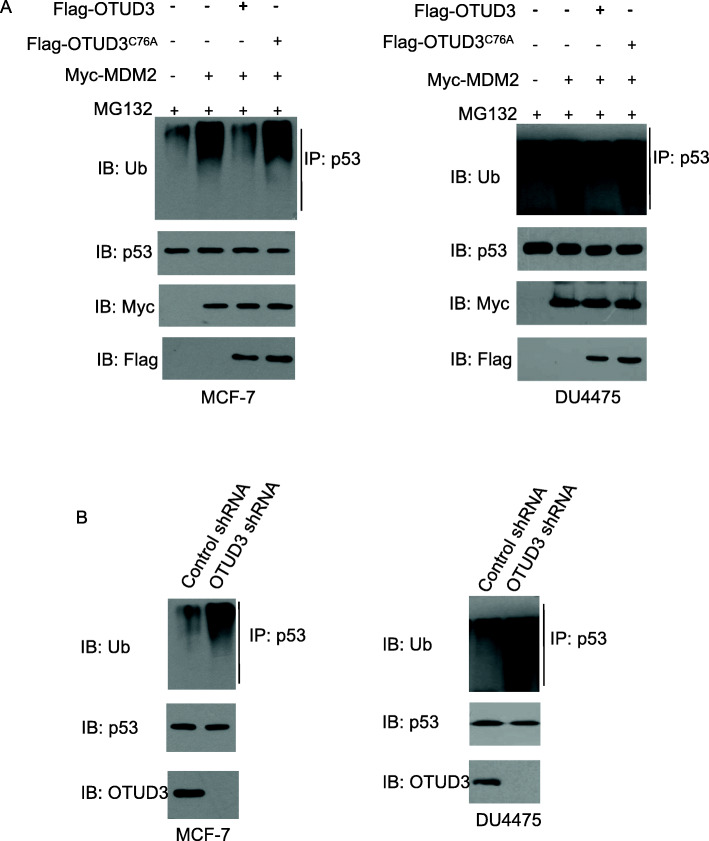
Regulation of p53 ubiquitination levels by OTUD3, OTUD3^C76A^ and Mdm2. MCF7 and DU4475 cells transfected with the indicated constructs (**a**) or stably expressing Ctrl shRNA or OTUD3 shRNA (**b**) were treated with MG132 before harvesting for the immunoprecipitation and immunoblot analyses with the indicated antibodies

As OTUD3 is a deubiquitinase, we examined whether OTUD3 deubiquitinates p53. To elucidate the molecular mechanism by which OTUD3 stabilizes p53, we determined whether OTUD3 directly controls the levels of p53 ubiquitination. As indicated in Fig. 5a, a high level of ubiquitinated p53 was found in the MCF7 and DU4475 cells transfected with Mdm2 (lane 2); however, p53 ubiquitination was significantly abrogated by OTUD3 expression (comparison of lanes 3 and 2). Notably, the enzyme activity of the mutant OTUD3^C76A^ lost its ability to deubiquitinate p53 (Fig. 5a, lane 4), indicating that p53 stabilization by OTUD3 requires deubiquitinating enzymatic activity. In contrast, the OTUD3 downregulation by shRNA increased p53 ubiquitination in the MCF7 and DU4475 cells (Fig. 5b). Collectively, these data demonstrate that OTUD3 negatively regulates p53 ubiquitination in breast cancer cells and plays an important role in the balance between p53 ubiquitination and deubiquitination. We speculate that the balance between the Mdm2-mediated ubiquitination and OTUD3-mediated deubiquitination of p53 is critical for p53 stabilization.

### OTUD3 inhibits cell proliferation

**Fig. 6:**
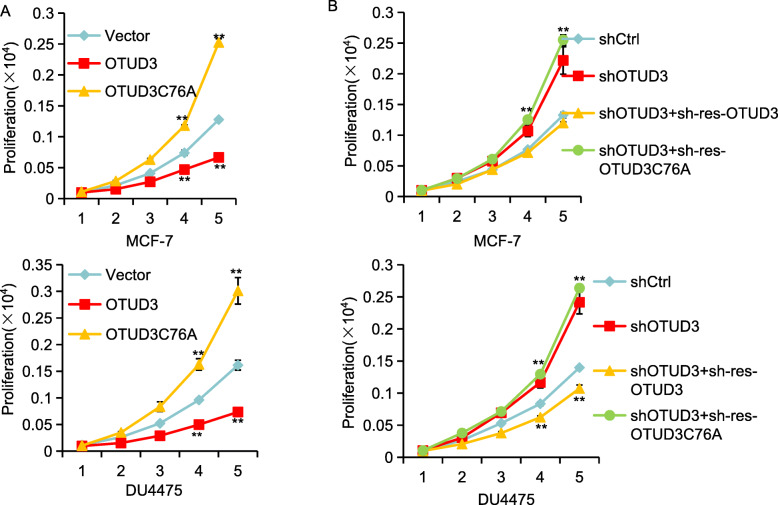
OTUD3 inhibits cell proliferation. **a** MCF7 and DU4475 cells transfected with OTUD3 and OTUD3^C76A^ were seeded in 96-well plates. Cell proliferation was measured by MTS assay at the indicated time. Data were analyzed using two-way ANOVA. Data are shown as the mean ± SEM of three independent experiments. **b** OTUD3 was knocked down by shRNA, and the OTUD3 and OTUD3^C76A^ levels were restored without shRNA interference for proliferation detection. The data are presented as the mean ± standard deviation of three independent experiments. *p < 0.05, **p < 0.01

P53 stabilization is crucial for its suppression of cell growth and apoptosis. To investigate the biological role of OTUD3, we first examined its effect on cell proliferation. We compared the proliferation rates of MCF7 and DU4475 cells stably transfected with OTUD3 and OTUD3^C76A^ with those of negative control cell lines using an MTS proliferation test kit. The results showed that cell proliferation slowed after OTUD3 transfection and accelerated after OTUD3^C76A^ transfection (Fig. 6a). Compared to the control cells, knockdown of endogenous OTUD3 by shRNA in the MCF7 and DU4475 cells increased the cell proliferation rate. However, the DU4475 cells proliferated at a significantly slower rate after OTUD3 expression was restored. OTUD3 could rescue the accelerated cell proliferation caused by OTUD3 knockdown, but the inactive enzyme mutant OTUD3^C76A^ could not inhibit the accelerated cell proliferation, indicating that regulation of the effect of OTUD3 on cell proliferation depends on its ubiquitinase activity (Fig. 6b). These data suggest that OTUD3 can inhibit BC cell proliferation.

### OTUD3 induces apoptosis in BC cells and inhibits colony formation

**Fig. 7:**
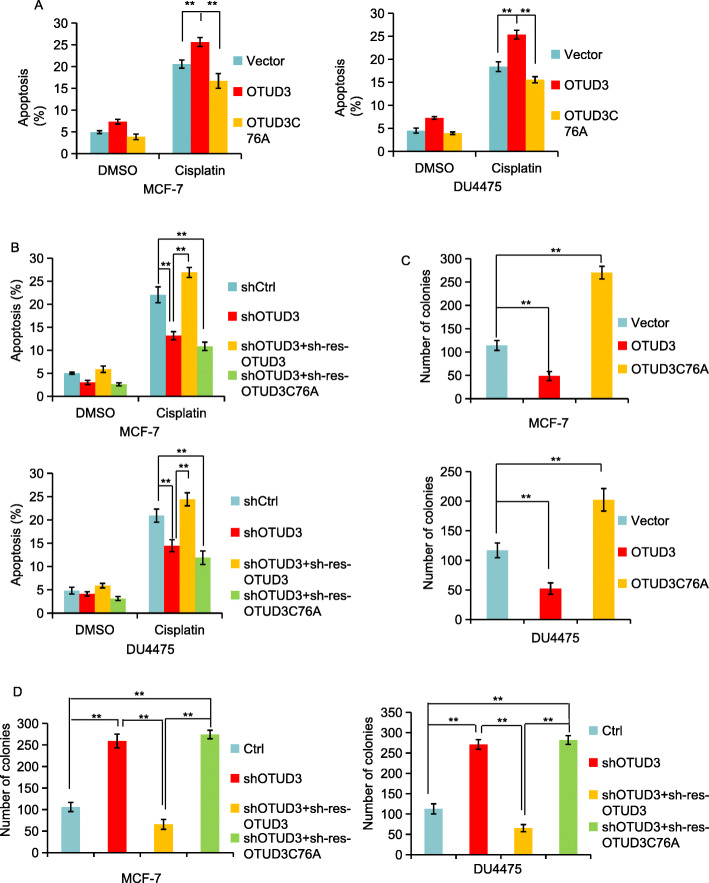
OTUD3 induces apoptosis in BC cells and inhibits colony formation. **a** Cell apoptosis detection: negative control and OTUD3 and OUUD3^C76A^ vectors were transfected into MCF7 and DU4475 cell lines. The cells were then subjected to an apoptosis detection. **b** OTUD3 knockdown by shRNA and OTUD3 and OTUD3^C76A^ restoration without shRNA interference for cell apoptosis detection. **c**-**d** Cell clone formation detection: OTUD3 negatively regulates cell clone formation in MCF7 cells and DU4475 cells. The data are presented as the mean ± standard deviation of three independent experiments. *p < 0.05, **p < 0.01

Chemotherapy is an important method for the treatment of BC. P53-mediated pathways can be activated by genotoxic compounds, such as cisplatin chemotherapeutic compounds, leading to cell cycle arrest and cell death [39]. We treated BC cells with the chemotherapy drug cisplatin (10 mM, 24 h) and detected apoptosis. The results showed that the OTUD3-transfected cells were significantly more sensitive to cisplatin-induced apoptosis than the negative control cells (*p <* 0.01); however, the sensitivity of the transfected OTUD3^C76A^ cells was significantly decreased (*p <* 0.01) (Fig. 7a). Compared with the negative control cells, the decreased OTUD3 protein levels increased the resistance of the tumor cells to cisplatin-induced apoptosis (*p <* 0.01). The apoptosis rate was significantly increased when OTUD3 was restored to the levels of the cells not treated with shRNA (*p <* 0.01), but when the levels of OTUD3^C76A^ were restored in the cells, the apoptosis rate did not significantly change (Fig. 7b). These results suggest that OTUD3 has a certain response to chemotherapy-induced BC cell apoptosis and that this response depends on its deubiquitinase activity.

Subsequently, we examined the effect of OTUD3 on cell growth using a colony formation assay. The BC cells were infected with either a control vector or a vector encoding OTUD3 or OTUD3^C76A^ and cultured for 2 weeks. Strikingly, OTUD3, but not OTUD3^C76A^, strongly inhibited the number of colonies of BC cells (Fig. 7c). When OTUD3 was knocked down, the cell clone formation ability was enhanced, and when OTUD3 was restored, the clone formation ability was inhibited (Fig. 7d). However, the cell cloning ability was significantly enhanced after OTUD3 ^C76A^ overexpression.

## Discussion

Our experiment proved for the first time that OTUD3 is a tumor-suppressing DUB in BC. The online database analysis showed that BC patients with high OTUD3 and p53 expression have better RFS, thus revealing a potential prognostic biomarker of BC. In addition, the mRNA expression of OTUD3 was lower in BC tissue than in normal adjacent tissue and was unrelated to the staging or molecular type. The clinical sample study of the Qilu cohort further proved that the protein expression of OTUD3 in BC tissues was lower than that in adjacent tissues. OTUD3 expression in cancer tissues was independent of the molecular type and histological classification. Therefore, the absence of OTUD3 is associated with the occurrence of BC. As a tumor suppressor gene, OTUD3 may serve as a new biomarker of the occurrence and development of BC. Targeting the OTUD3 upstream and downstream pathways may be a useful therapeutic strategy because BC cells may have lost the expression of such tumor-suppressing DUBs.

Functional p53 prevents the progression of cancer by increasing growth inhibition in the form of apoptosis, senescence and/or autophagy [40]. Deubiquitination is a major mechanism that stabilizes p53 and induces apoptosis. Regulation of p53 ubiquitination and deubiquitination in BC is of great interest but remains poorly understood. Our study proves for the first time that downregulation of OTUD3 in clinical BC samples highly coincides with downregulation of p53. OTUD3 can directly interact with and stabilize p53 through deubiquitination in BC cells. The N-terminal of OTUD3 contains an OTU domain that directly participates in the binding of the T2 and T3 sequences of p53. Decreased OTUD3 expression may be an important mechanism underlying the loss of TP53 function in breast cancer cells carrying WT TP53 alleles. Both breast cancer cell lines used in this study were p53 wild-type BC cells—the luminal BC cell line MCF-7 and the TNBC cell line DU4475. The functional experiments using BC cells further confirmed OTUD3 anticancer function. OTUD3 supplements enzymes that can regulate p53 by deubiquitination and participates in protein-protein interactions in BC. Thus, OTUD3 is of great significance.

The major causes of death from breast cancer are relapse, drug resistance and metastasis, which are highly related to dysregulation of the MDM family [41–43]. The MDM family comprises the E3 ligase MDM2 and its close homologue MDM4 (alternatively termed MDMX). MDM2 is a vital regulator of tumor suppressor p53 activity in the breast [7, 8, 44] and has been identified as an independent prognostic biomarker in BC [45]. The complex between MDM2 and p53 is largely formed by the interaction between the N-terminal domain of MDM2 and the N-terminal transactivation (TA) domain of p53 (residues 15–29) [46, 47]. The N-terminal domain of p53 contains the main Mdm2 binding site. The finding that OTUD3 potentially binds the N-terminus of p53 may suggest that both Mdm2 and OTUD3 compete for the same binding site in p53, possibly explaining the observed effects of OTUD3 overexpression and knockdown on p53 ubiquitination and p53 levels in cells. This spatially separates MDM2 from p53, resulting in the stabilization of the p53 protein and allowing p53 to regulate gene transcription, leading to p21 and BAX expression, cell cycle arrest, and/or cell death.

We found that OTUD3 deletion is generally associated with the obliteration of WT p53 in BC, suggesting that OTUD3 loss may be selected by tumors to disrupt the p53 pathway. Although our findings reveal an important mechanism by which p53 can be stabilized by direct deubiquitination and imply that OTUD3 might function as a tumor suppressor in vivo through the stabilization of p53, many questions remain unanswered. Our study found that the OTUD3 mRNA level was not related to DNA methylation through an online database; thus, the reason for the decrease in OTUD3 expression in BC remains to be further explained. In addition, the effect of OTUD3 on other key regulators in the p53 pathway must also be examined.

## Conclusions

OTUD3 is a cancer-suppressing DUB in BC that can positively regulate the function and stability of p53. The OTUD3-p53 interaction may be involved in the formation of BC.

## Supplementary information

**Additional file 1.**

## Data Availability

The datasets generated during the current study are available from the corresponding author upon reasonable request.

## Abbreviations

BC: Breast cancer
RFS: Recurrence-free survival
mRNA: Messenger ribonucleic acid
ER: Estrogen receptor
PR: Progesterone receptor
HER-2: Human epidermal growth factor receptor-2
IHC: Immunohistochemistry
WB: Western blot
DUBs: Deubiquitinating enzymes
CHX: Cycloheximide
CoIP: Coimmunoprecipitation
CTD: C-terminal regulatory domain
DBD: DNA-binding core domain
NLS: Nuclear localization signaling domain
TAD: Transcription-activation domain
DMSO: Dimethyl sulfoxide
FBS: Fetal bovine serum
SDS: Sodium dodecyl sulfate
SPSS: Statistical Product and Service Solutions
