# Supplementary material for: Tumor suppressor OTUD3 induces growth inhibition and apoptosis by directly deubiquitinating and stabilizing p53 in invasive breast carcinoma cells
Source: BMC Cancer. 2020 Jun 22;20:583. doi: 10.1186/s12885-020-07069-9 (PMC7310228; doi:10.1186/s12885-020-07069-9)
Supplement: Supplementary file 1 — Additional file 1. [file 12885_2020_7069_MOESM1_ESM.ppt]

## Slide 1
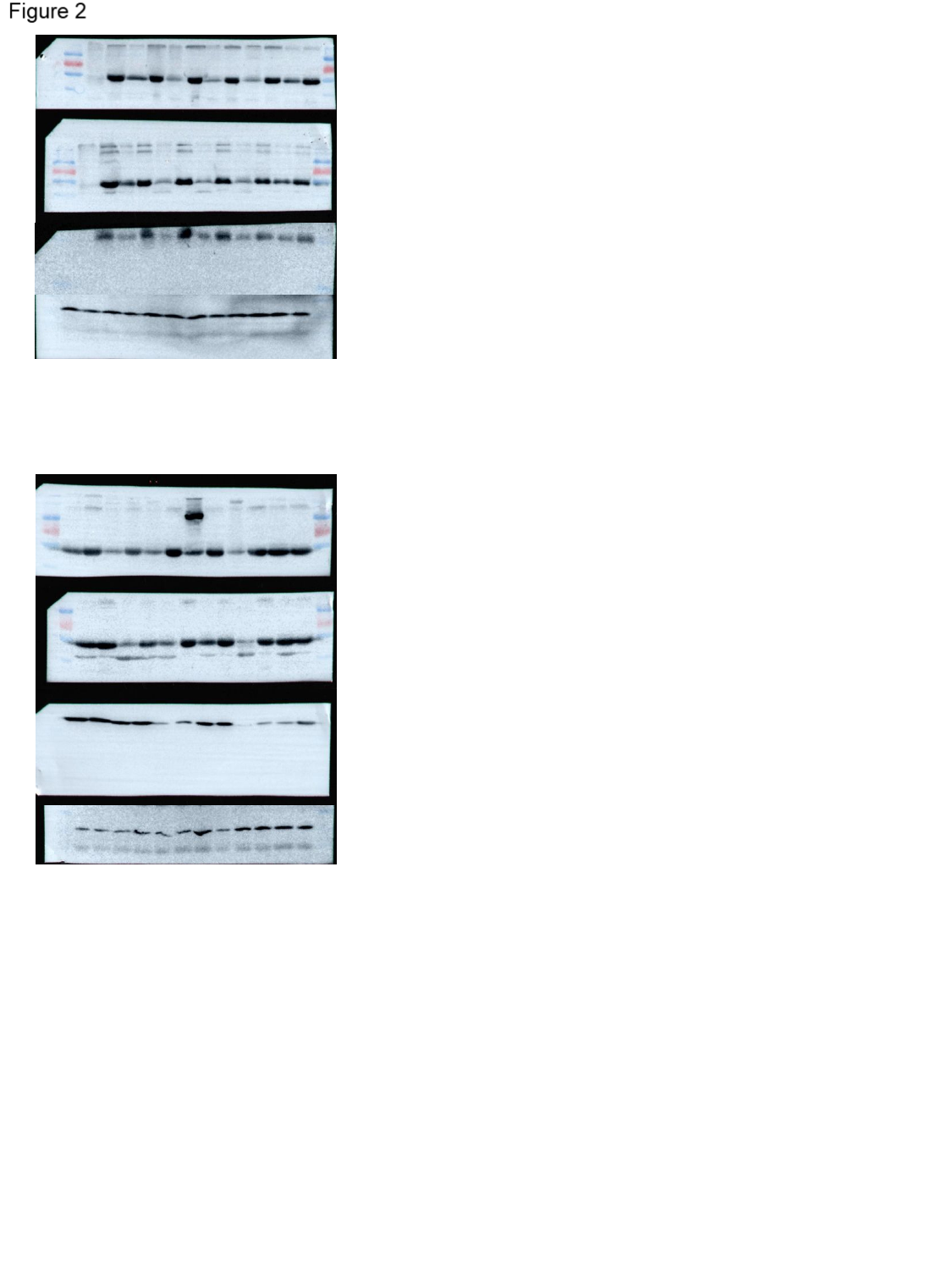

## Slide 2
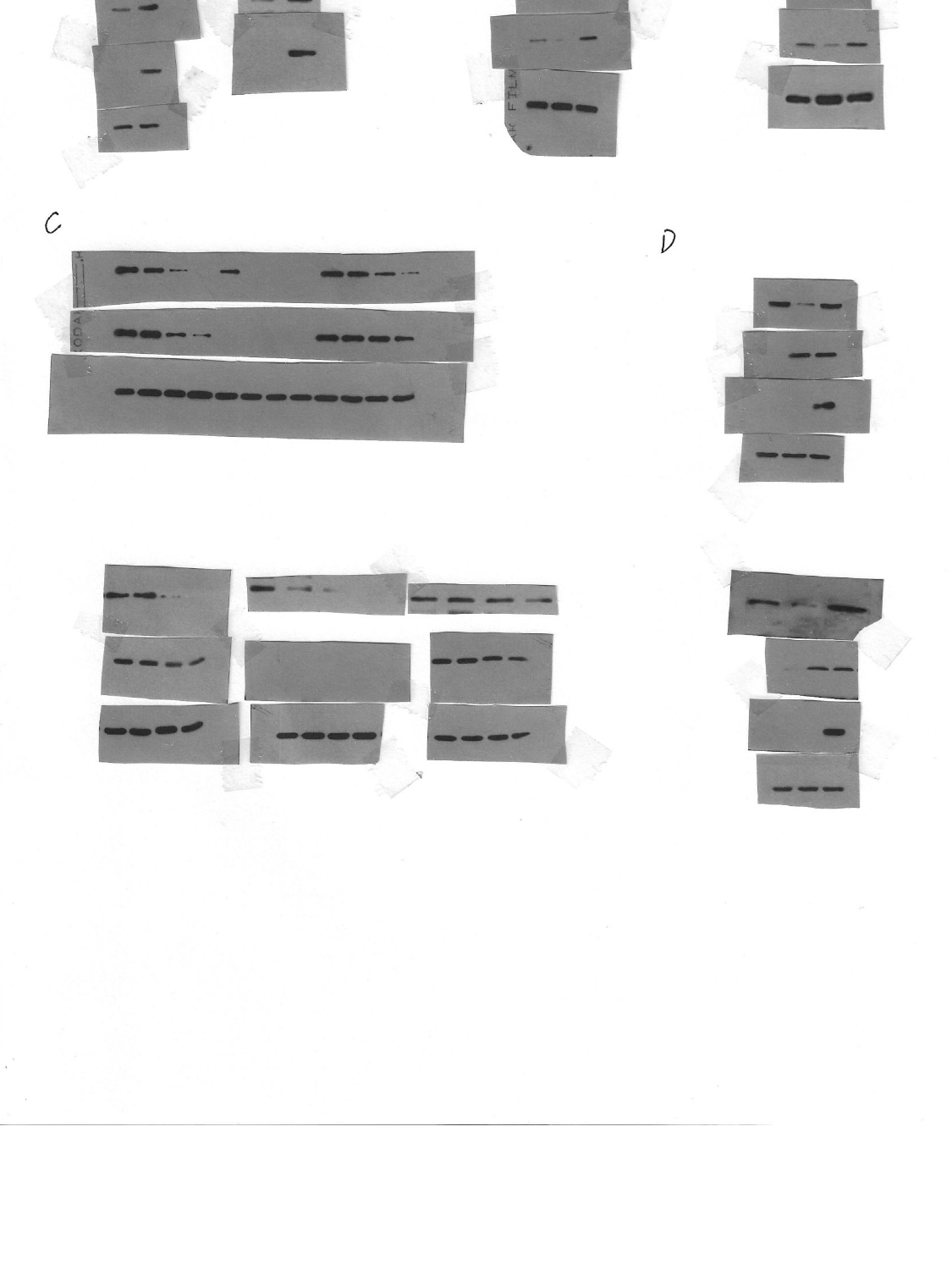

#

## Slide 3
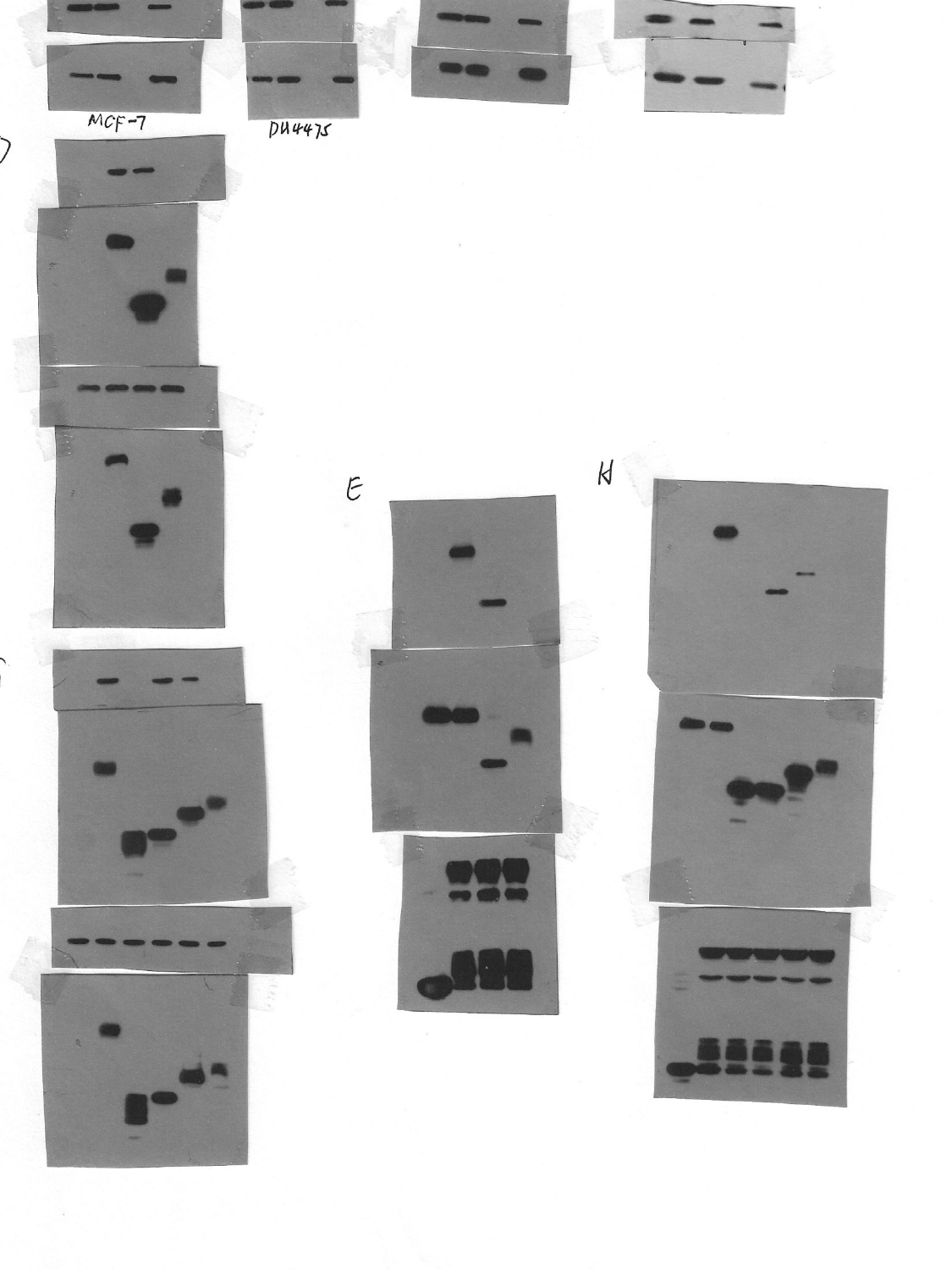

## Slide 4
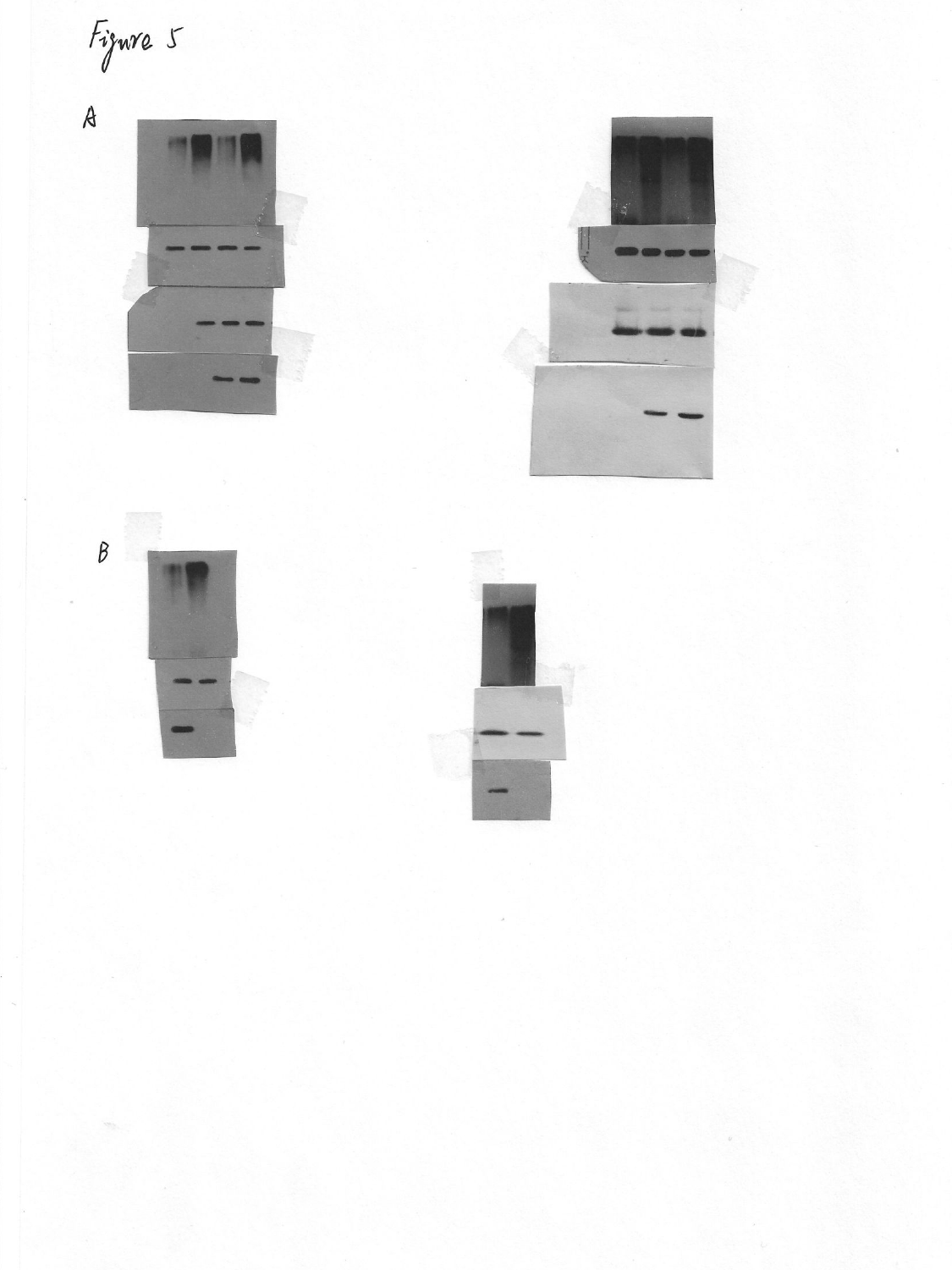

#

## Slide 5
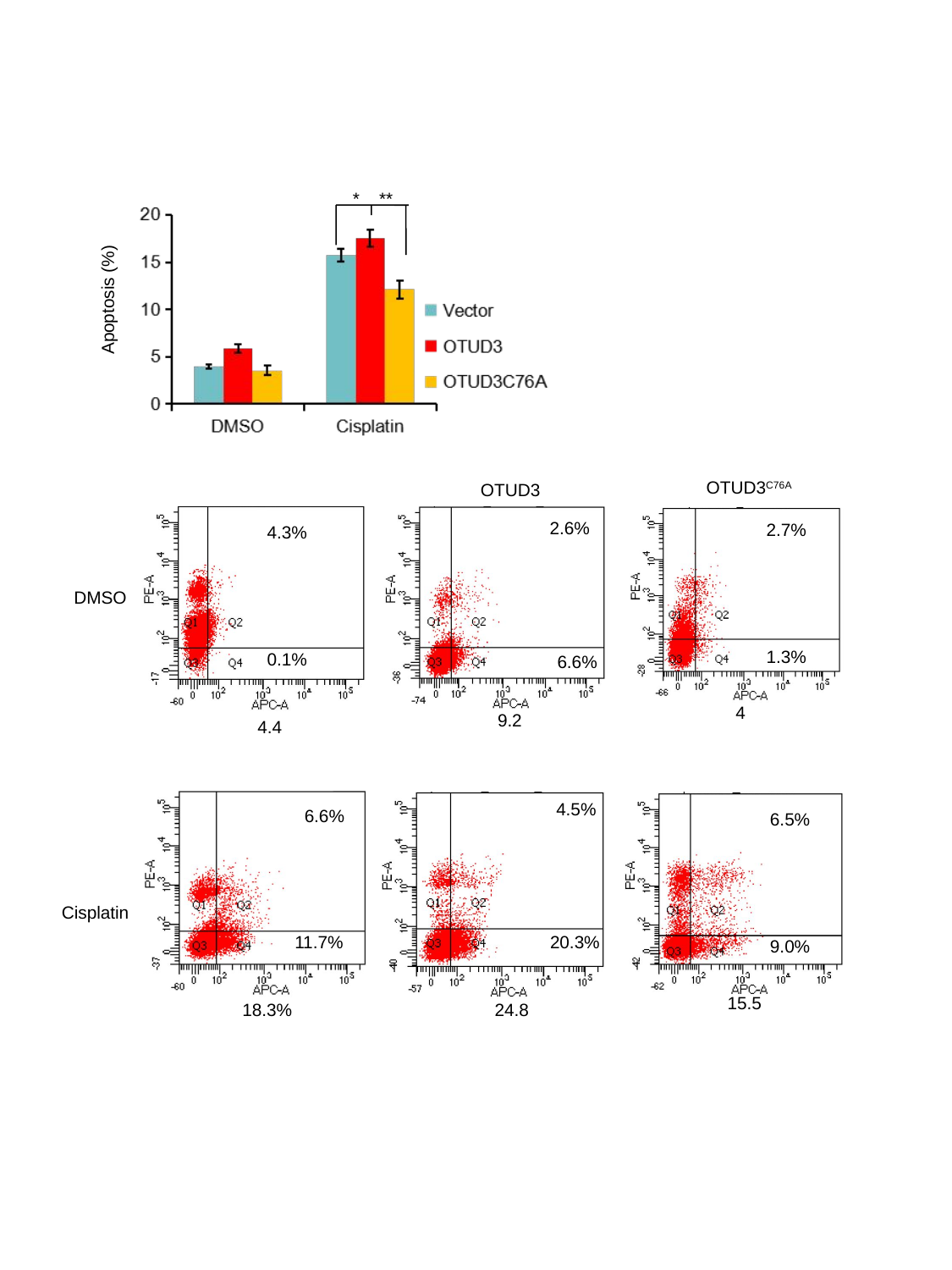

*
**
Apoptosis (%)
OTUD3C76A
OTUD3
 2.6%
2.7%
1.3%
4.3%
0.1%
DMSO
6.6%
4
9.2
4.4
 4.5%
 6.6%
11.7%
6.5%
9.0%
Cisplatin
20.3%
15.5
24.8
18.3%

## Slide 6
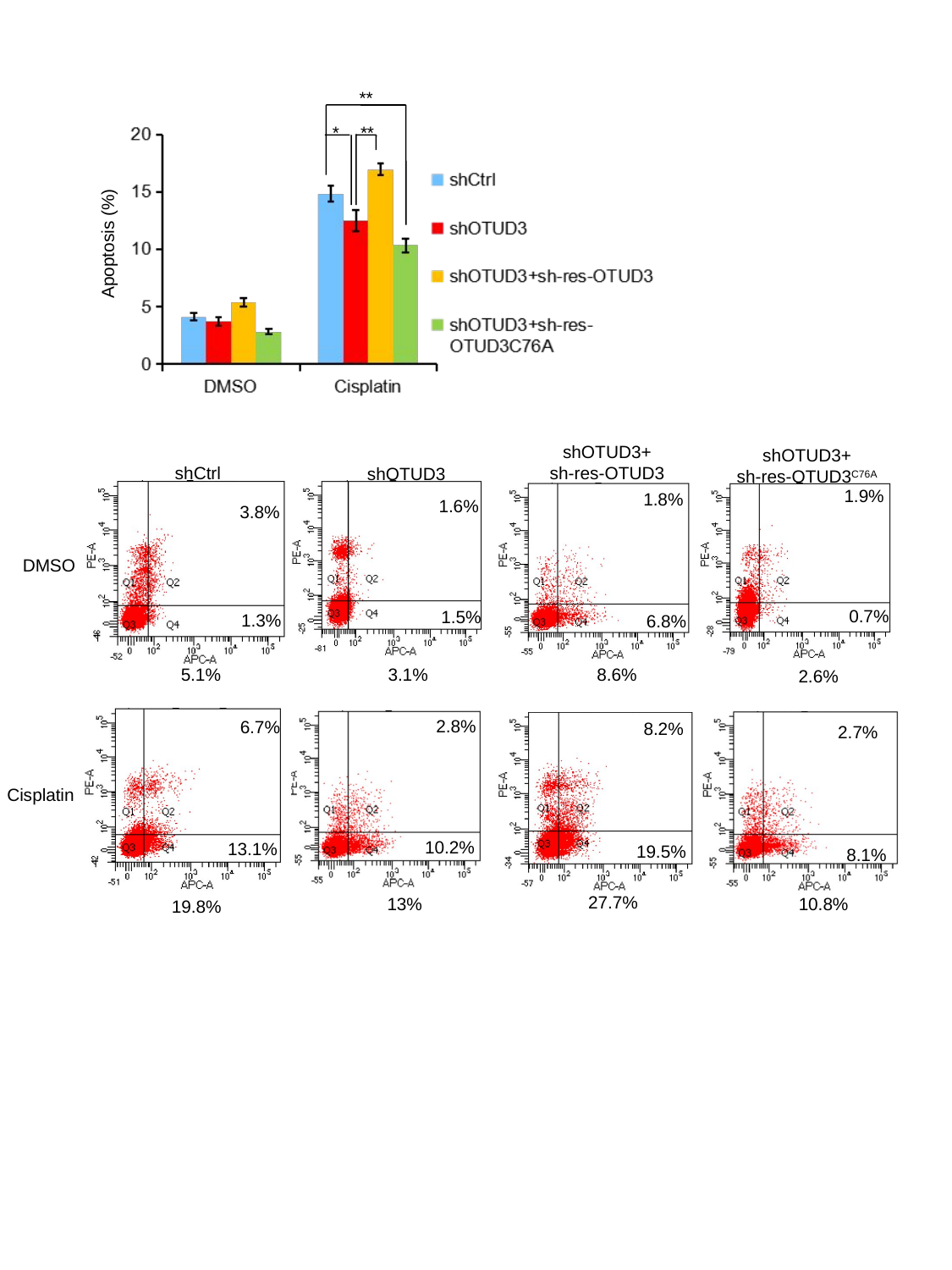

**
**
*
Apoptosis (%)
shOTUD3+
sh-res-OTUD3
shOTUD3+
sh-res-OTUD3C76A
shCtrl
shOTUD3
1.9%
1.8%
1.6%
3.8%
DMSO
0.7%
1.5%
1.3%
6.8%
5.1%
3.1%
8.6%
2.6%
2.8%
6.7%
8.2%
2.7%
Cisplatin
10.2%
13.1%
19.5%
8.1%
27.7%
13%
10.8%
19.8%

## Slide 7
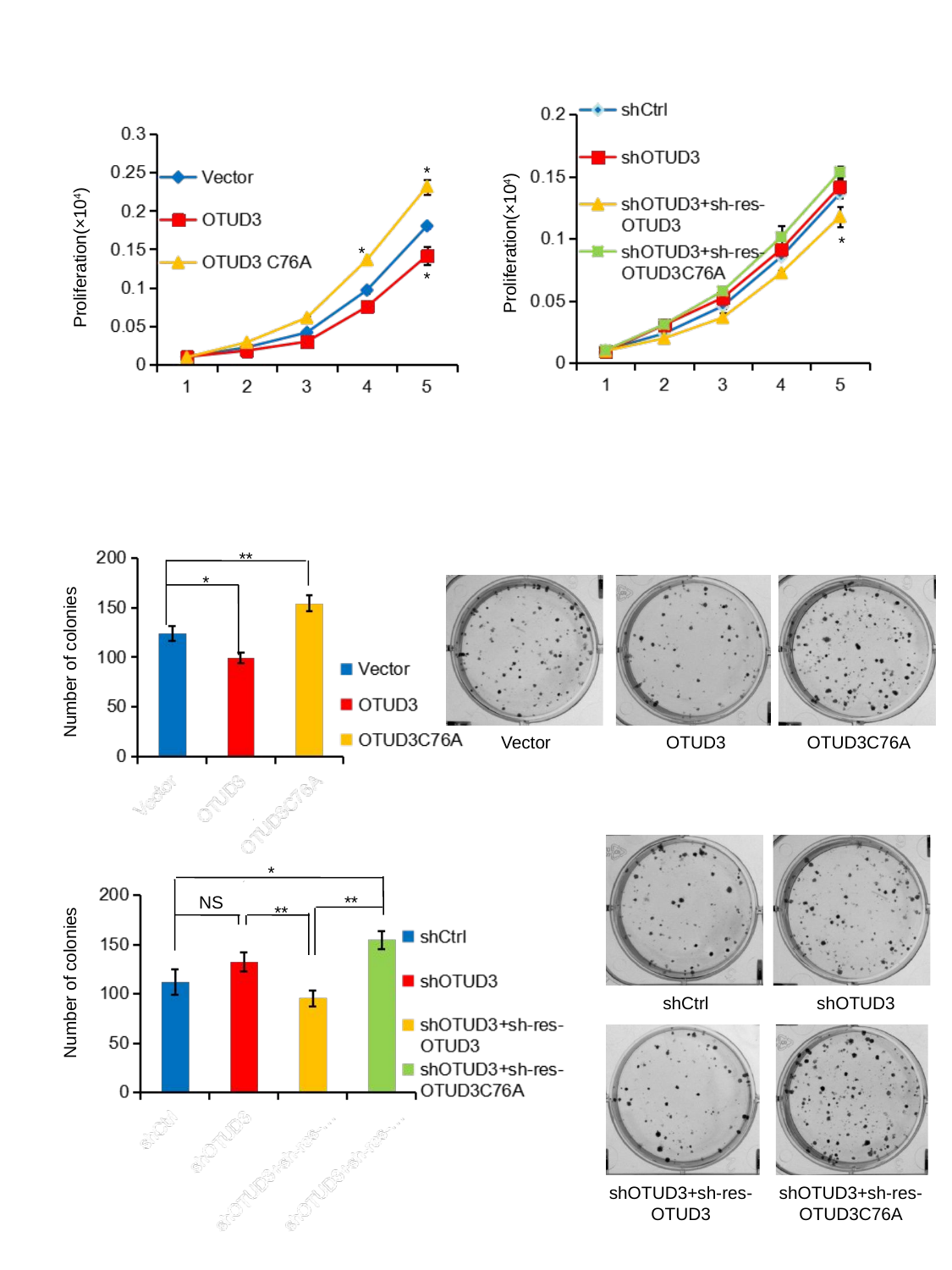

Proliferation(×104)
*
Proliferation(×104)
*
*
*
Number of colonies
**
*
Vector
OTUD3
OTUD3C76A
Number of colonies
*
NS
**
**
shCtrl
shOTUD3
shOTUD3+sh-res-OTUD3
shOTUD3+sh-res-OTUD3C76A
